# Draft genome assembly and annotation of *Haematococcus lacustris* strain Liv1, an industrial astaxanthin-producing microalga

**DOI:** 10.1128/mra.00591-25

**Published:** 2025-10-20

**Authors:** Michael J. Lashbrook, Jeremiah A. Adesanya, Jayson Talag, Kathleen Lail, Raymond Lee, Anna M. Lipzen, Jie Guo, Jerry Jenkins, Alan Kuo, Christa Pennacchio, Igor V. Grigoriev, Rhona K. Stuart, Christopher S. Ward

**Affiliations:** 1 Department of Biological Sciences, Bowling Green State University 1888, Bowling Green, Ohio, USA; 2 Arizona Genomics Institute, University of Arizona 8041, Tucson, Arizona, USA; 3 US Department of Energy Joint Genome Institute 118576, Berkeley, California, USA; 4 HudsonAlpha Institute for Biotechnology 69980, Huntsville, Alabama, USA; 5 Department of Plant and Microbial Biology, University of California Berkeley 1438, Berkeley, California, USA; 6 Physical and Life Sciences Directorate, Lawrence Livermore National Laboratory 4578, Livermore, California, USA; Rochester Institute of Technology, Rochester, New York, USA

**Keywords:** microalgae, bioproducts, genome assembly, genome annotation

## Abstract

*Haematococcus lacustris* is a ubiquitous unicellular green alga with industrial bioproduct applications, namely, as feedstock for natural astaxanthin. We report the annotated 291.5 Mbp genome for *H. lacustris* Liv1 to support future algal research in the areas of carotenoid biosynthesis and crop protection.

## ANNOUNCEMENT

*Haematococcus lacustris* (class Chlorophyceae, order Chlamydomonadales, family Haematococcaceae) is a microalgal species that is cultivated industrially for astaxanthin, a carotenoid with strong antioxidant properties produced under various abiotic stress conditions (1). Genomic insights into improving astaxanthin production are limited by the availability of only two sequenced strains (i.e., *H. lacustris* NIES-144 and *H. lacustris* SAG192.80; [2–4]). As demand for astaxanthin increases, having genome resources that reflect the functional and ploidy diversity of the *Haematococcus* genus will become increasingly valuable.

The strain *H. lacustris* Liv1 was selected for industrial cultivation due to its high biovolume and astaxanthin content per cell. For genome sequencing, the culture was obtained from Lawrence Livermore National Lab (USA) and maintained in Bristol’s modified medium (5) under 14/10 h light/dark photocycles (80 μE m^−2^ s^−1^) at 25°C. To create a pooled transcript library, sub-cultures were transferred to fresh Bristol’s media, heterotrophic media (Tris-acetate-phosphate, [6]), and reddening media (7) incubated in normal light, as well as Bristol’s media in dark (0 μE m^−2^ s^−1^). Nucleic acid extraction using Quick-DNA/RNA Miniprep Kit (Zymo) was performed at the Arizona Genomics Institute.

Genomic DNA was sequenced at the Joint Genome Institute (JGI) using PacBio and Illumina sequencing, following similar protocols to (8). For PacBio, 10 µg of genomic DNA was sheared to 30–50 kb using Megaruptor 3 (Diagenode). Sheared DNA was treated with exonuclease, DNA damage repair enzyme mix, and end-repair/A-tailing mix; ligated with overhang adapters using SMRTbell Express Template Prep Kit 2.0 (PacBio); and purified with AMPure PB Beads (PacBio). Individual libraries were size-selected using 0.75% agarose gel cassettes with Marker S1 and High Pass protocol on the BluePippin (Sage Science). PacBio Sequencing primers were then annealed to the SMRTbell template library, and sequencing polymerase was bound using Sequel II Binding kit 1.0. The prepared SMRTbell template libraries were then sequenced on a Pacific Biosciences Sequel II sequencer using 8M v.1 SMRT cells and v.1.0 sequencing chemistry with 1 × 900 sequencing movie run times. For Illumina, 100 ng of genomic DNA was sheared to 500 bp using the Covaris LE220 and size-selected with SPRI using TotalPure NGS beads (Omega Bio-tek). Fragments were treated with end repair, A-tailing, and ligation of Illumina-compatible adapters (IDT) using the KAPA-HyperPrep kit (KAPA Biosystems). For transcriptome sequencing, stranded cDNA libraries were generated using the Illumina Truseq Stranded RNA LT kit. mRNA was purified from 1 µg of total RNA using magnetic beads containing poly-T oligos. mRNA was fragmented and reverse transcribed using random hexamers and SSII (Invitrogen) followed by second-strand synthesis. The fragmented cDNA was treated with end pair, A-tailing, adapter ligation, and eight cycles of PCR. The prepared Illumina libraries were quantified using KAPA Biosystems’ Next-Generation Sequencing Library qPCR Kit (Roche) and run on a Roche LightCycler 480 Real-Time PCR instrument. The quantified library was then multiplexed with other libraries, and the pool of libraries was then prepared for sequencing on the Illumina NovaSeq 6000 sequencing platform (2 × 150 indexed run) using NovaSeq XP v.1 reagent kits and an S4 flow cell.

Genome assembly involved reads being assembled with MECAT2 (9) and polished with ARROW v.2.0 (Pacific Biosciences). Homozygous single-nucleotide polymorphisms and insertion–deletions were corrected in the raw assembly consensus using ~53× of Illumina reads. To facilitate gene annotation, the transcriptome (430 million paired-end reads) consisted of two parts: (i) Illumina reads were assembled with Trinity v.2.11; and (ii) PacBio reads were clustered into Consensi with IsoSeq v.9.0. Default parameters were used except where otherwise noted. Functional annotation using the JGI annotation (10) pipeline resulted in 21,367 protein-coding gene models (Table 1). The average estimated protein length is 422 amino acids, with 7.14 exons per gene. Genome assembly size and gene count are comparable to other published *Haematococcus* genomes (https://phycocosm.jgi.doe.gov/chlorophyceae). This genome will support continued optimization of microalgal bioproduction and contribute foundational data for genome-informed strain selection and development.

**TABLE 1:** Summary of *H. lacustris* Liv1 genome assembly and annotation statistics

Characteristics	*H. lacustris* Liv1
Genome assembly size (Mbp)	291.5
Sequencing read coverage depth	297×
No. of scaffolds	1,648
Scaffold N50 (kb)	360
Scaffold L50	222
No. of gaps	0
GC content (%)	60.5
No. of genes	21,755
No. of protein-coding genes	21,367

## Data Availability

The *H. lacustris* v.1.2 draft genome is provided through the Phycocosm portal (11) from the U.S. Department of Energy Joint Genome Institute at https://phycocosm.jgi.doe.gov/Haeplu1. Whole genome sequence data are available in the National Center for Biotechnology Information under BioProject accession number PRJNA1080846, BioSample accession number SAMN40207212, and Sequence Read Archive Project accession numbers SRX28835866 and SRX28836051.
